# Identification of a novel GPR143 mutation in a large Chinese family with isolated foveal hypoplasia

**DOI:** 10.1186/s12886-021-01905-7

**Published:** 2021-03-30

**Authors:** Xiying Mao, Mingkang Chen, Yan Yu, Qinghuai Liu, Songtao Yuan, Wen Fan

**Affiliations:** Department of Ophthalmology, The First Affiliated Hospital of Nanjing Medical University, Nanjing, 210029 China

**Keywords:** GPR143 mutation, Isolated foveal hypoplasia, OA1, Case report

## Abstract

**Background:**

Pathogenic variants of G-protein coupled receptor 143 (GPR143) gene often leads to ocular albinism type I (OA1) characterized by nystagmus, iris and fundus hypopigmentation, and foveal hypoplasia. In this study, we identified a novel hemizygous nonsense mutation in GPR143 that caused an atypical manifestation of OA1.

**Case presentation:**

We reported a large Chinese family in which all affected individuals are afflicted with poor visual acuity and foveal hypoplasia without signs of nystagmus. Fundus examination of patients showed an absent foveal reflex and mild hypopigmentation. The fourth grade of foveal hypoplasia and the reduced area of blocked fluorescence at foveal region was detected in OCT. OCTA imaging showed the absence of foveal avascular zone. In addition, the amplitude of multifocal ERG was reduced in the central ring. Gene sequencing results revealed a novel hemizygous mutation (c.939G > A) in GPR143 gene, which triggered p.W313X. However, no iris depigmentation and nystagmus were observed among both patients and carriers.

**Conclusions:**

In this study, we reported a novel nonsense mutation of GPR143 in a large family with poor visual acuity and isolated foveal hypoplasia without nystagmus, which further expanded the genetic mutation spectrum of GPR143.

## Background

GPR143, also known as the ocular albinism type 1 (OA1) gene, encodes a 7TM G-protein coupled protein and is exclusively expressed by pigment cells. The mutation of GPR143 leaded to OA1, an X-linked type of albinism, which results in nystagmus, impaired visual acuity and foveal hypoplasia [1].

The form of albinism in OA1 patients affects the eye, especially iris and fundus, but the pigmentation of hair and skin is usually normal. The early sign of OA1 is mostly nystagmus that usually appears within 6 months after birth [2]. Since iris and fundus hypopigmentation is not obvious among Asians, OA1 is usually misdiagnosed as the congenital idiopathic nystagmus [3–5]. Up to date, more than one hundred mutations in the GPR143 gene have been identified in OA1 in the world, but GPR143 variants without nystagmus have been rarely reported [6].

In this study, we reported and characterized a large Chinese family, in which all the affected individuals are afflicted with poor visual acuity and foveal hypoplasia as the predominant manifestations, while no sign of nystagmus was detected. Gene sequencing results revealed the disease-causing gene to GPR143 with a novel point mutation (c.939G > A) in exon 8. Together, our results presented this previously unidentified mutation of GPR143 that caused the isolated foveal hypoplasia without nystagmus.

## Case presentation

**Fig. 1:**
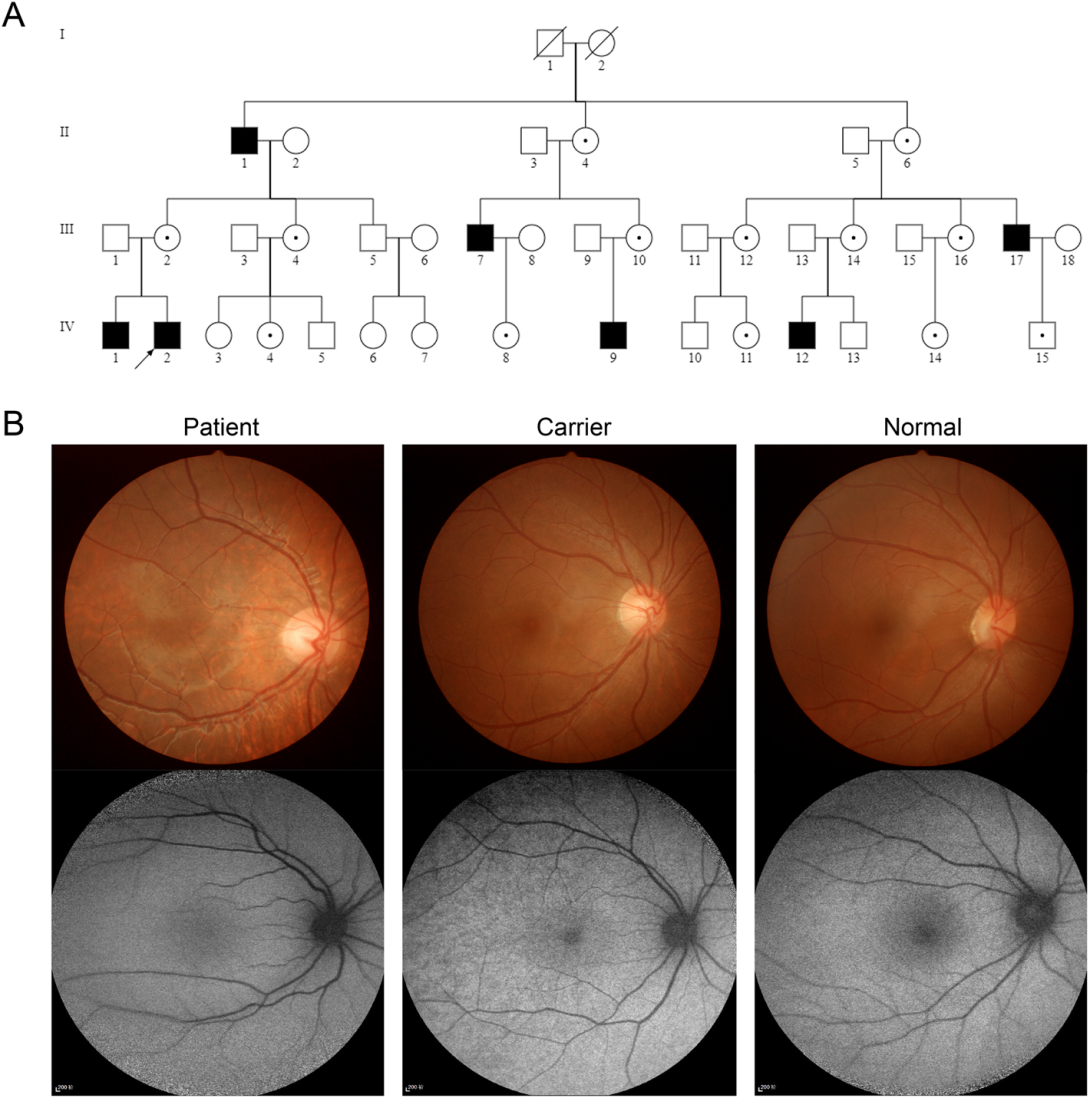
The pedigree structure and fundus imaging. **a** The pedigree structure of a Chinese family with the isolated foveal hypoplasia. The proband is marked with an arrow. **b** Representative fundus photos from patient, carrier and normal individual

We identified a large, three-generation Chinese family with 25 living members, among whom 6 members were patients (Fig. 1A). All affected individuals were males, suggesting the inheritance pattern of the disease in the family was typical X-linked recessive.

The proband was misdiagnosed as amblyopia before he came to our hospital. The visual acuity of right and left eyes was 0.15 and 0.25, respectively, at his first visit. Fundus examination showed an absent foveal reflex and hypopigmentation in the entire retina (Fig. 1B). No iris depigmentation and nystagmus were observed.

**Fig. 2:**
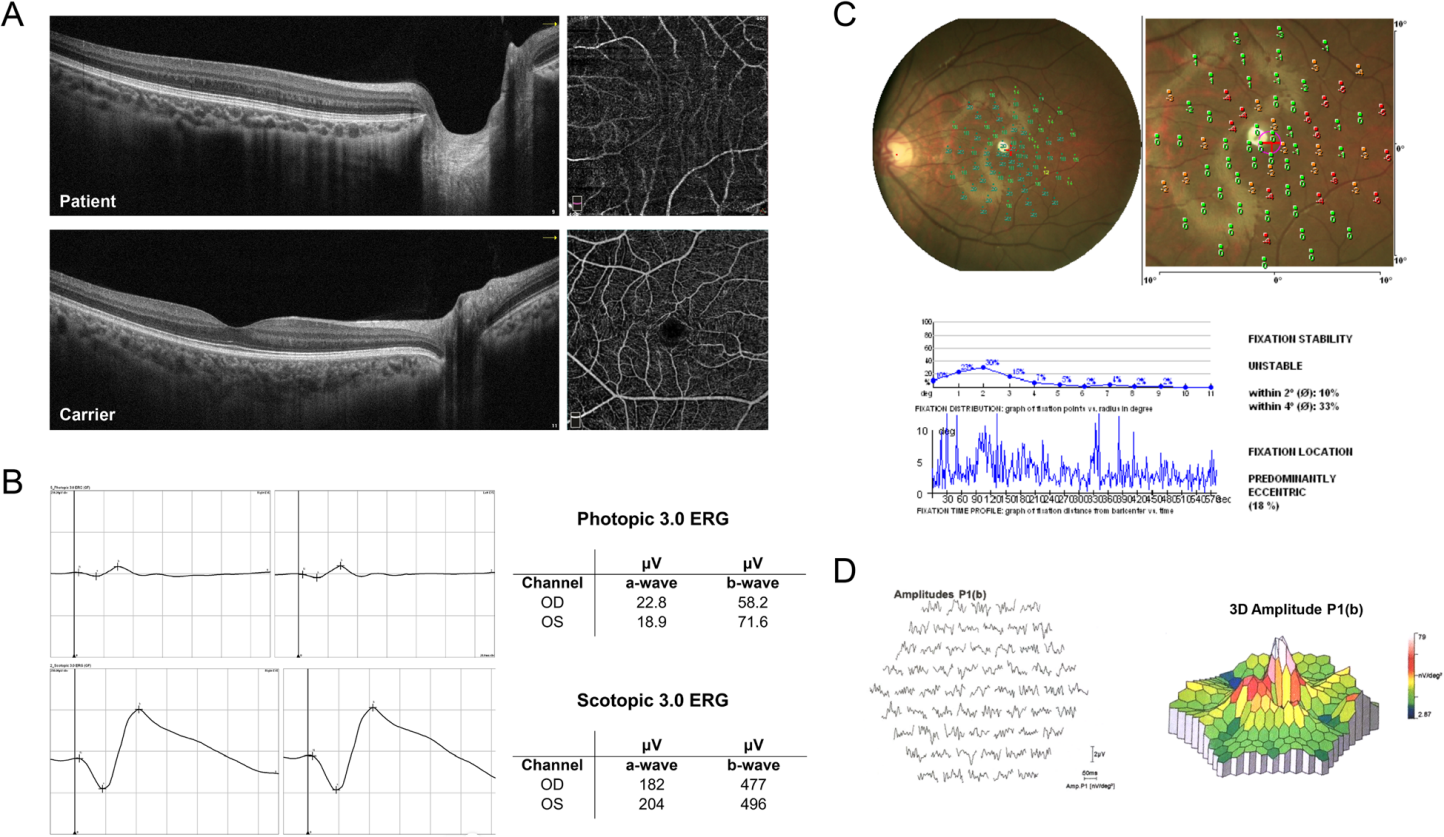
The ophthalmological examinations. **a** Representative imaging of OCT and OCTA of patient and carrier. **b** Full-field ERG result of the proband. **c** Microperimetry result of the proband. **d** Multi-focal ERG result of the proband

OCT imaging showed grade 4 foveal hypoplasia. Grade 4 foveal hypoplasia is characterized by absence of foveal pit, absence of extrusion of plexiform layers and absence of lengthening of outer segments of photoreceptors [7]. OCT-angiography (OCTA) imaging showed the absence of foveal avascular zone (FAZ) (Fig. 2A). Fundus autofluorescence imaging (AFI) revealed the reduced area of blocked fluorescence at foveal region, indicating the macular pigment was severely affected (Fig. 1B). In addition, the amplitude of b-wave was attenuated in the full-field ERG recording, which is correlated with the dysfunction of photoreceptor respectively (Fig. 2B). VEP result was normal, without the typical pattern of VEP asymmetry showed in ocular albinism which correlates with the severity of albinism [8]. A poor fixation was revealed in microperimetry (The fixation stability within 4°: 33%), but the mean sensitivity was normal (Fig. 2C). In multifocal ERG, the amplitude was reduced in the central ring and flattened slope of amplitudes was observed from central to peripheral ring, indicating the depressed macular function (Fig. 2D).

Other affected individuals and obligate female carriers were also characterized clinically. Grade 4 foveal hypoplasia was identified in all patients, but not in carriers. Mild fundus hypopigmentation was observed in both patients and carriers. All family members were free from nystagmus and iris depigmentation (Table 1).

**Table 1:** Clinical features for patients and carriers in this study

ID#	Patient/Carrier	Gender	Age	Mutation	Foveal hypoplasia	Fundus hypopigmentation	Nystagmus	Iris hypopigmentation
II:1	Patient	male	65	Hemizygous	Yes	Obvious	No	No
III:2	Carrier	female	36	Heterozygous	No	Mild	No	No
III:7	Patient	male	32	Hemizygous	Yes	Obvious	No	No
III:12	Carrier	female	29	Heterozygous	No	Mild	No	No
III:14	Carrier	female	26	Heterozygous	No	Mild	No	No
III:16	Carrier	female	25	Heterozygous	No	Mild	No	No
III:17	Patient	male	22	Hemizygous	Yes	Obvious	No	No
IV:2	Patient	male	5	Hemizygous	Yes	Obvious	No	No
IV:4	Carrier	female	7	Heterozygous	No	Mild	No	No
IV:8	Carrier	female	5	Heterozygous	No	Mild	No	No
IV:9	Patient	male	2	Hemizygous	Yes	Obvious	No	No
IV:11	Carrier	female	0.5	Heterozygous	No	Mild	No	No
IV:12	Patient	male	6	Hemizygous	Yes	Obvious	No	No
IV:15	Carrier	female	3	Heterozygous	No	Mild	No	No

**Fig. 3:**
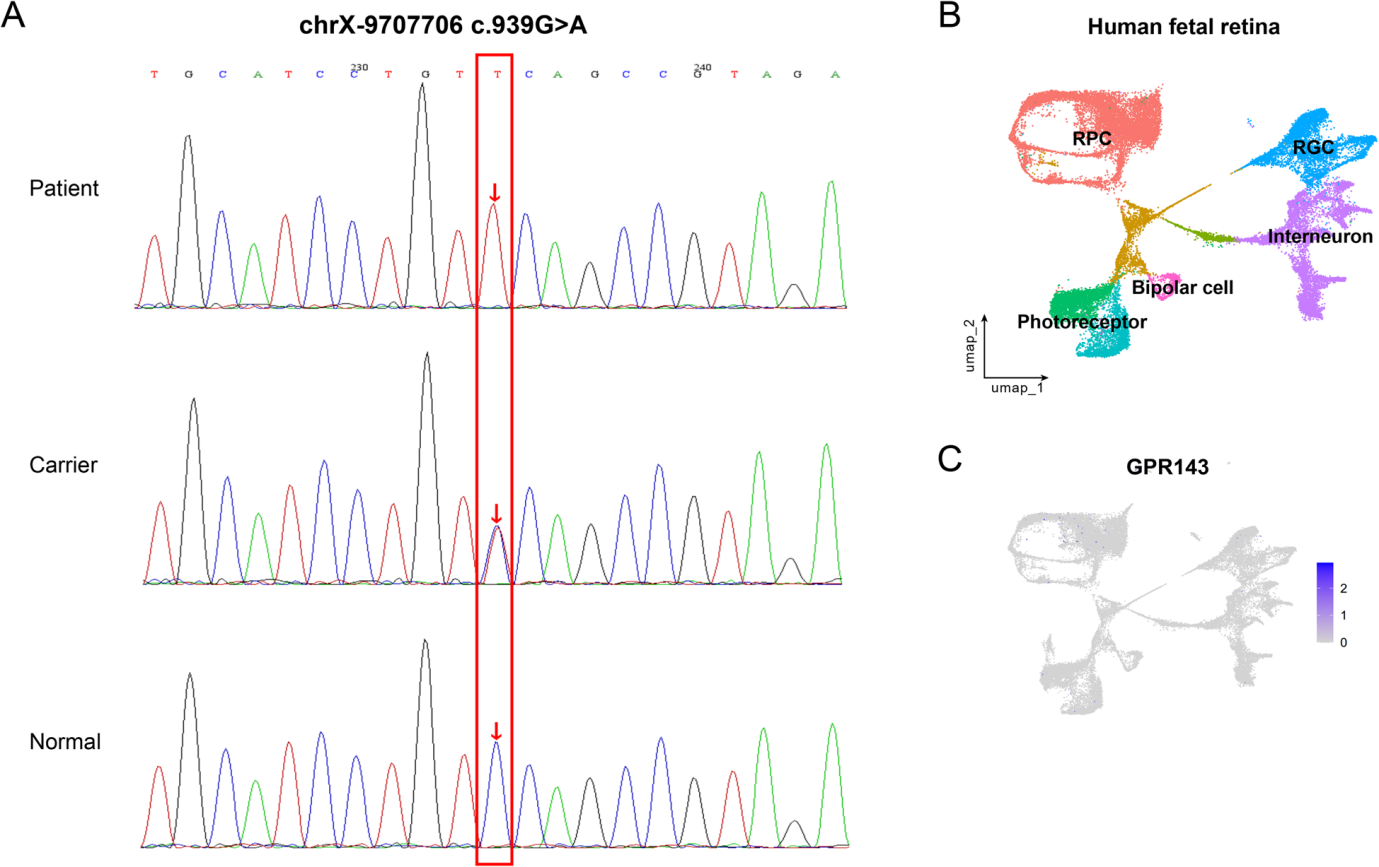
Identification of a novel mutation of GPR143. **a** DNA sequencing showing a G to A transition at nucleotide 939 of GPR143 in patient and carrier. **b** GPR143 expression in the published single-cell transcriptome data of fetal retina

Furthermore, we screened for mutations on the genes from a specific hereditary eye disease enrichment panel based on targeted exome capture technology (Gencap) and next-generation sequencing (MyGenostics, Beijing). A hemizygous GPR143 variant (c.939G > A, nucleotide 939 in exon 8 is changed from guanine to adenine) was identified in the proband. This mutation belongs to the nonsense mutation (p.W313X). No cases of this mutation were retrieved in Human Gene Mutation Database. Sanger sequencing confirmed the mutation in all patients and showed a heterozygous mutation at this locus in the female carriers (Fig. 3A).

A glass prescription was given for full-time wear and the proband was instructed to return every year for follow up. Due to the irregular glass wear during 2018, corrected visual acuity was dropped at the third visit. After education, vision in the new glasses was improved and stable at around 0.5 in each eye at his last visit, suggesting the importance of the early-stage intervention of refractive errors to avoid amblyopic development (Table 2). Consistent with the result at the first visit, fundus examination showed no foveal landmark in either eye. Taken together, a diagnosis of isolated foveal hypoplasia was given.

**Table 2:** Visual acuity of proband during the 3-year follow-up

	OD	OS
2017-08-02	+ 1.50DS/− 2.00 DC X 175 → **0.4**	+ 2.00DS/− 2.50 DC X 180 → **0.5**
2018-02-20	+ 1.00DS/− 2.00 DC X 85 → **0.4**	+ 1.25DS/− 2.00 DC X 85 → **0.6**
2019-04-22	+ 2.50DS/− 1.75 DC X 170 → **0.2**	+ 3.00DS/− 2.00 DC X 180 → **0.3**
2020-03-21	+ 1.25DS/− 1.75 DC X 175 → **0.4**	+ 1.50DS/− 1.75 DC X 180 → **0.7**

## Discussion

In this study, we identified a novel nonsense mutation c.939G > A (p.W313X) in exon 8 of GPR143 among patients with impaired visual acuity and foveal hypoplasia. More than 100 mutations of GPR143 have been detected in family and sporadic cases, which is commonly associated with OA1 characterized by nystagmus, ocular hypopigmentation and foveal hypoplasia. However, no symptom of nystagmus was observed in our cases, indicating variable severities may be attributed by different locations of mutation.

GPR143 encodes a pigment cell specific G protein-coupled receptor protein which is expressed mainly in melanosomes [9]. Sequencing analysis revealed the mutation of a G to A transition at nucleotide 939 of GPR143. The mutation leads to the substitution of tryptophan to a stop codon at codon 313 (p.W313X) which causes the loss of C-terminal. Subsequently, the motif (p.329–330) containing melanosomal membrane localization signal is destroyed, which dampens the function of intracellular melanosome biogenesis [10, 11]. Consistently, we observed the mild hypopigmentation in the fundus imaging of patients. In addition, the mild hypopigmentation was also found in the fundus of carriers, possibly due to X chromosome inactivation [12]. In GPR143-knockdown melanocyte (including RPE), the number and movement of melanosome were significantly decreased, while GPR143 overexpression stimulated the production of melanin content in a dose-dependent manner [13, 14]. This dosage-dependent effect of GPR143 on pigment biogenesis was also demonstrated in other human genetic studies that carrier females showed patches of hypopigmentation of the fundus but did not have symptoms of foveal hypoplasia and usually have good visual acuity [15–17].

The combination of AFI and fundus photography indicated the significant reduction of macular pigment in patients, while carriers were affected to a lesser extent. The physiological FAZ was also lost at the foveal region of the patient showed in OCTA imaging. It is well-established that both the formation of FAZ and the accumulation of macular pigment are the contributing factors of foveal development [18]. However, the association of these fovea-related factors to GPR143 remains largely elusive.

The expression of GPR143 is found in pigment cells, including retinal pigment epithelium. However, in the published single-cell transcriptome data of human fetal retina [19], we cannot find the restricted expression of GPR143 in any of the developing retinal subtypes (Fig. 3B). Therefore, the result suggested a potential paracrine effect of GPR143 signaling in RPE on the neighboring foveal development. In previous studies, L-DOPA was demonstrated as the endogenous ligand of GPR143 [20]. L-DOPA/GPR143 mediates the negatively control the vascularization by increasing the secretion of PEDF and reducing VEGF production [21]. The vascular inhibition of GPR143 signaling may subsequently lead to foveal formation. As for the effect on macular pigment, increasing evidence have been shown that GPR143 activity may protect against both onset and progression of age-related macular degeneration (AMD) [22, 23]. Low macular pigment is a key risk factor for AMD, which indicates the potential association between GPR143 signaling and macular pigment [24]. Therefore, it could be postulated that GPR143 signaling can extrinsically promote the foveal formation via attenuating retinal vascularization and increasing the production of macular pigment.

## Conclusions

Foveal hypoplasia from GPR143 variants is usually associated with conditions, such as eye albinism and nystagmus. Isolated foveal hypoplasia has been rarely reported and the corresponding GPR143 mutation was unknown [6]. In our study, we have identified a novel mutation of GPR143 in Chinese family with nystagmus-free foveal hypoplasia, which further expanded the genetic mutation spectrum. Further investigation of the function of GPR143 in foveal development may help to elucidate the mechanisms of the disease and molecular targets for potential clinical treatment.

## Data Availability

The datasets generated and analyzed during the current study are available in the SRA repository (https://www.ncbi.nlm.nih.gov/sra). The accession number is PRJNA707130.

## Abbreviations

GPR143: G-protein coupled receptor 143
OA1: Ocular albinism type 1
OS: Outer segment
OCT: Optical coherence tomography
OCTA: Optical coherence tomography angiography
FAZ: Foveal avascular zone
AFI: Fundus autofluorescence imaging
ERG: Electroretinogram
VEP: Visual evoked potential
RPE: Retinal pigment epithelium
PEDF: Pigment epithelium-derived factor
VEGF: Vascular endothelial growth factor
AMD: Age-related macular degeneration
